# Using concept mapping to evaluate knowledge structure in problem-based learning

**DOI:** 10.1186/s12909-015-0496-x

**Published:** 2015-11-27

**Authors:** Chia-Hui Hung, Chen-Yung Lin

**Affiliations:** 1 Department of Rehabilitation, Jen-Teh Junior College of Medicine, Nursing and Management, No. 79–9, Sha-Luen Hu, Xi-Zhou Li, Hou-Loung Town, Miaoli, Taiwan; 2 Graduate Institute of Science Education, National Taiwan Normal University, No. 88, Ting-Jou Rd., sec. 4, Taipei, Taiwan

**Keywords:** Concept map, Knowledge structure, Occupational therapy, Problem-based learning

## Abstract

**Background:**

Many educational programs incorporate problem-based learning (PBL) to promote students’ learning; however, the knowledge structure developed in PBL remains unclear. The aim of this study was to use concept mapping to generate an understanding of the use of PBL in the development of knowledge structures.

**Methods:**

Using a quasi-experimental study design, we employed concept mapping to illustrate the effects of PBL by examining the patterns of concepts and differences in the knowledge structures of students taught with and without a PBL approach. Fifty-two occupational therapy undergraduates were involved in the study and were randomly divided into PBL and control groups. The PBL group was given two case scenarios for small group discussion, while the control group continued with ordinary teaching and learning. Students were asked to make concept maps after being taught about knowledge structure. A descriptive analysis of the morphology of concept maps was conducted in order to compare the integration of the students’ knowledge structures, and statistical analyses were done to understand the differences between groups.

**Results:**

Three categories of concept maps were identified as follows: isolated, departmental, and integrated. The students in the control group constructed more isolated maps, while the students in the PBL group tended toward integrated mapping. Concept Relationships, Hierarchy Levels, and Cross Linkages in the concept maps were significantly greater in the PBL group; however, examples of concept maps did not differ significantly between the two groups.

**Conclusions:**

The data indicated that PBL had a strong effect on the acquisition and integration of knowledge. The important properties of PBL, including situational learning, problem spaces, and small group interactions, can help students to acquire more concepts, achieve an integrated knowledge structure, and enhance clinical reasoning.

## Background

Competent practitioners in the health care professions are developed not only through the acquisition of the biomedical knowledge and clinical skills necessary to provide high-quality, effective services but also through the development of an integrated knowledge structure in an active and personal way [1–4]. A knowledge structure, which is the set of cognitive processes used by clinical practitioners in the diagnosis of patients, is characterized by an elaborate, highly integrated framework of related concepts [3, 5]. Knowledge structure theory implies that both learners and experts can be influenced by their prior knowledge or underlying knowledge structures when producing diagnostic hypotheses and participating in problem-solving activities [6–8]. If educators can employ better ways to facilitate the development of an integrated knowledge structure than the rote memorization of facts or procedural practice, then it is likely that they will be able to promote the development of greater competence in the health care professions.

Problem-based learning (PBL) originated in Canada in the 1960s in response to dissatisfaction with the traditional didactic teaching curriculum in medical education and a perceived need for reform in the education of medical students [9, 10]. The PBL approach, an innovative teaching and learning method utilized in medical education, may provide greater challenge and motivation by utilizing real-life scenarios to engage students by activating their prior knowledge, increasing understanding of basic science concepts, and organizing compartmental knowledge to construct a rich, elaborate, and well-integrated knowledge structure, in order to foster learning and transfer knowledge from the theoretical to the clinical context [11, 12]. Furthermore, elaborately designed problems can stimulate self-directed learning strategies, team participation skills, information retention, and reasoning and problem-solving skills that will be available to the student after graduation [13–15]. If basic science and declarative knowledge can actually be converted into skills or “demonstrations”, then higher levels of performance or competency can be achieved [2, 16]. Although PBL can enhance problem-solving and clinical reasoning skills in the health care professions [17], previous research on its superiority to the lecture-based learning (LBL) approach in the acquisition of basic science knowledge has produced inconsistent findings [18–21]. Research has also indicated that an integrated knowledge structure, rather than compartmentalized knowledge, is a prerequisite for successful problem-solving [2, 17]; however, little supportive empirical evidence has been reported to show that the development of a knowledge structure is enhanced by PBL [2].

Since the original purpose of PBL was to promote deeper content learning [22], it is important to develop insights into students’ knowledge structures; however, the objective assessment methods often employed in PBL focus on bits of factual knowledge and techniques in medical problem solving and tend to value formal and routine procedural reasoning [7, 8, 23]. It is not sufficient to develop the knowledge and problem-solving skills in PBL, for it is important to develop higher-order thinking skills and meaningful learning with organized concepts, as opposed to the mere collection of facts [11, 24]. Studies of health care professionals suggest that concept mapping can provide a clear representation of a student’s knowledge structure [14, 15, 25, 26]. Concept mapping is a schematic device for organizing and representing a set of concepts embedded in a framework of propositions by graphically illustrating the complex processes or relationships among relevant concepts within a given subject domain [13, 27–30]. Concept mapping, which was developed by Novak and Gowin based on the Ausubelian Association Theory of meaningful learning [30, 31], can be used to show the whole knowledge structure of students. The process of learning refers to the anchoring of new ideas or concepts in previously acquired knowledge in a non-arbitrary way, thereby allowing students to differentiate concepts, integrate them into an existing knowledge structure, and form intentional effort linkages among isolated concepts by themselves [14, 26, 32]. In this view, lower-order concepts are linked from linear to departmental, and integrated under higher-order concepts through integrative reconciliation and progress differentiation. In integrative reconciliation, meaningful learning makes it easier for students to identify the similarities or differences between concepts, thus enabling them to take the relevant concepts and construct a superordinate concept [31, 33]. In progress differentiation, higher-order concepts are differentiated into more elaborate and hierarchical levels in the knowledge structure [34, 35]. Thus, concept mapping has the potential to assess the dynamic reasoning about concept relationships in students’ knowledge structures during PBL [36]. Observing the structure and details can help teachers to identify difficulties in reasoning and improve students’ higher order thinking skills [37].

Concept mapping may serve as an effective, feasible, and acceptable tool for evaluating and monitoring students’ learning in PBL [36–38]. Its effectiveness can be assessed with two approaches. First, concept mapping can show the formation of a knowledge structure from the basic structure to a depiction of the hierarchy and relationships among concepts [39, 40]. Second, it can also show the high degree of coherence and connectedness within the knowledge structure that is related to the holistic morphology of construction patterns [41]. Both approaches concentrate on understanding the structure of knowledge to show the depth of thinking required in clinical reasoning [42]. These two approaches, which deal with the inner hierarchy and morphological features, were employed together in the current study to reveal the expansion and evolution of knowledge structures as a result of PBL.

## Methods

### Study aim

This study compared the learning methods of PBL and LBL on students’ development of knowledge structures. The research question was: What patterns of concepts and their differences in the knowledge structures between the PBL and LBL groups can be identified from the use of concept maps for evaluating students’ learning achievement? A quasi-experimental method design was employed. Concept mapping was used to evaluate the effects of learning outcomes, including the patterns of concept mapping and the knowledge structure.

### Participants

The study was conducted as part of the course “Assessment and Management of Brain Function: Perspectives of Occupational Therapy” in an occupational therapy program at a medical college in the Taipei area. A total of 52 occupational therapy undergraduates in their third year (20 to 21 years of age) participated in the study. None of the participants had previously been exposed to PBL or concept mapping. The students were divided into four small groups of 13 students each. The researcher randomly assigned each student to one of the four groups according to the student’s registration number. Two of the groups were assigned to the PBL experimental group, and the other two, to the LBL control group. GPower statistical software was used to calculate a sample size sufficient for a power of 0.8 as suggested by Howell [43]. Given the mean difference, standard deviation, and effect size noted in the results, the sample size of 26 participants in each group was appropriate. Although no explicit IRB approval was sought, since it was not required for educational research in Taiwan in 2010, ethical approval was granted by three occupational therapy professors outside the research team. The general principles of the Declaration of Helsinki were followed, identifying information in the data was removed to ensure anonymity, and informed consent was given by the participants.

### Preparation of the PBL Program

The PBL program used in this study was based on the Maastrich 7-step PBL method [10]. During the PBL sessions, students were asked to work in collaboration with group members to analyze two cases of cognitive disability in occupational therapy. The PBL sessions took place 1 day per week over 6 weeks, and each case lasted for 3 weeks. The students followed the 7-step process to work through the PBL problems. This allowed them to explore the symptoms and clinical problems of the patients with cognitive disabilities, demonstrate clinical reasoning, make appropriate intervention decisions, and finally develop management plans. The PBL scenarios in the study presented four key features that were designed to trigger motivation, connect to prior knowledge, organize content knowledge, and evoke new learning.

**The first feature** was designed to trigger motivation. The initial chapter, a half-page case scenario, presented the dilemma faced by clients in their daily occupations. The scenario contained ordinary descriptions without professional terminology in order to facilitate students’ motivation and to elicit their concepts of the clinical problems.

**The second feature** was designed to connect concepts to prior knowledge. The subsequent two chapters in the PBL, each of which was one page in length, provided the medical history, occupational performance, and laboratory data of the clients. The aim of this stage was to develop extensive knowledge connections and a conceptual hierarchy. Students were encouraged to use professional terminology to describe symptoms and occupational therapy problems, choose an appropriate method of evaluation to identify the problems, and decide on their interventions.

**The third feature** was the problem space. The last chapter, one page in length, briefly described the key interventions of occupational therapy and the prognosis of the client. Several intervention techniques were roughly described as cues for possible solutions, and problem spaces were open for students to organize content knowledge, engage in discussions with group members, and develop their intervention plans.

**The fourth feature** was designed to link affective and attitudinal issues to facilitate learning. Ethical issues were provided in the scenarios to evoke further discussion among the students in the area of medical humanities.

### Data collection

Students in both groups attended basic biomedical classes on brain function for 3 hours per week over 10 weeks. The two groups were then separately exposed to the different learning methods for 6 weeks; PBL for the PBL group, and lectures for the LBL group. The LBL group met for 3 hours once per week, during which they continued their lectures and practiced with evaluation tools, and further practice or discussion was allowed after the classes.

Before students made their concept maps, they received two hours of instruction on concept mapping. Concept mapping was carried out after each problem case in order to understand how their knowledge changed as they gradually became involved in the discussion, and students handed in their maps the following week. Two sets of 10 terms related to cognitive disability in occupational therapy were given, as follows: executive function, experience, neuropsychological evaluation, aging, compensatory strategies, routine, tabletop activity, LOTCA, problem solving, abstract thinking, attention, higher-order thinking ability, self-awareness, culture, physical evaluation, A-ONE, cognitive retraining, personality, habit, and computer-based exercise. The participants were asked to prepare their own concept maps and encouraged to add any terms that they felt necessary to complete them. Two concept maps were developed by each student, and in the end, 104 concept maps were collected.

### Analytical process and methods

**Fig. 1:**
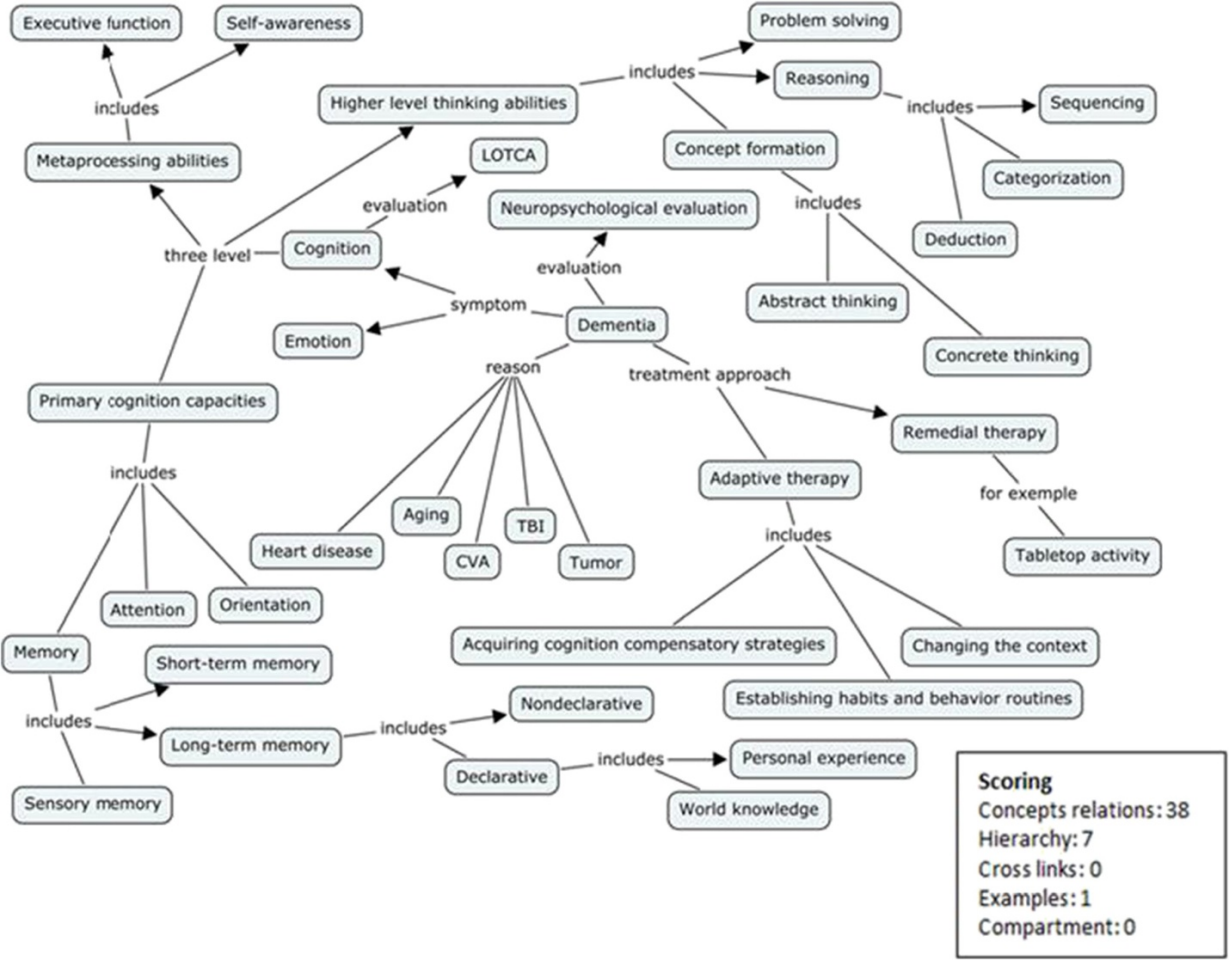
A map made by a participant and its scoring

The analysis was conducted in two steps. The first step focused on developing a global view of the knowledge structure, and the second examined the detailed connections among concepts in the concept maps. In the first step, the maps were examined to determine their morphology, including the whole structure and the component blocks, by two teachers of occupational therapy. The two teachers identified three types of morphology: isolated mapping, departmental mapping, and integrated mapping. Chi square tests were conducted to test the homogeneity of the two groups for the three types. In the second step, a quantitative scoring protocol devised by Novak & Gowin [30] was employed to investigate the students’ concept maps. The numbers of each of the four scoring parameters, the relationships among the concepts, the levels on the map, the cross linkages, and the examples were calculated by the two teachers. Independent-samples *t*-tests were performed to compare the differences in the knowledge structures in terms of the concept mapping scoring parameters between the PBL and LBL groups. The intra-class correlation coefficient (ICC) was calculated to estimate the inter-rater reliability for each scoring parameter. Figure 1 shows a concept map made by one participant. In this map, the term ‘dementia’ was initially chosen as the core for the map, and then the other terms related to dementia, such as symptoms, evaluations, and interventions, were added to develop the concept map. The map depicts a rather complicated knowledge structure with multiple blocks connected by appropriate linkage words. Each block contains a number of given and additional terms that demonstrate meaningful hierarchical relationships within the knowledge structure. A corresponding example, desktop activity, was added to the end of “remedial therapy” to show the participant’s suggestion for an intervention. No cross linkage was found among the blocks on this map, however, indicating that the participant failed to clarify connections.

## Results

### Content validity

The two case scenarios and their learning objectives were developed by the authors and were subsequently reviewed by three experts in occupational therapy to determine the consistency between the case content and learning objectives. The Pearson coefficient was 0.92, indicating a high degree of correlation between the case scenarios and the PBL learning objectives.

### Reliability

Reliability was established by the scores that two trained raters awarded on 29 concept maps drawn by the participants. The ICC coefficient for interrater reliability of the concept mapping scores indicated good agreement in concept relationships of 0.99 (95 % CI, 0.95–0.99); in the hierarchy, of 0.96 (95 % CI, 0.84–0.96); in levels, of 0.92 (95 % CI, 0.72–0.93); and in cross linkages, of 0.95 (95 % CI, 0.79–0.95) (Table 1).

**Table 1:** 95 % CI for inter-rater reliability on the scoring parameters

		95 % CI			
Scoring	ICC (2, 1)	Lower	Upper	*F*	*p*
Concept relationships	0.99	0.95	0.99	74.90	0.000^*^
Hierarchy levels	0.96	0.84	0.96	24.37	0.000^*^
Cross linkages	0.92	0.72	0.93	12.91	0.000^*^
Examples	0.95	0.79	0.95	18.37	0.000^*^

*p < 0.05; 95 % CI = 95 % Confidence Interval; ICC = Intra-Class Correlation Coefficient

### Analysis based on morphology

Generally speaking, constructing a concept map begins with the definition of the topic that the concept map is to address. Then key concepts related to the knowledge structure of the map are identified and listed. Those concepts are then considered and sorted in terms of their inclusiveness from general through moderate to specific. After all, a concept map is built to reveal the intended knowledge structure. Three major categories were identified based on the morphology of the maps.

**Fig. 2:**
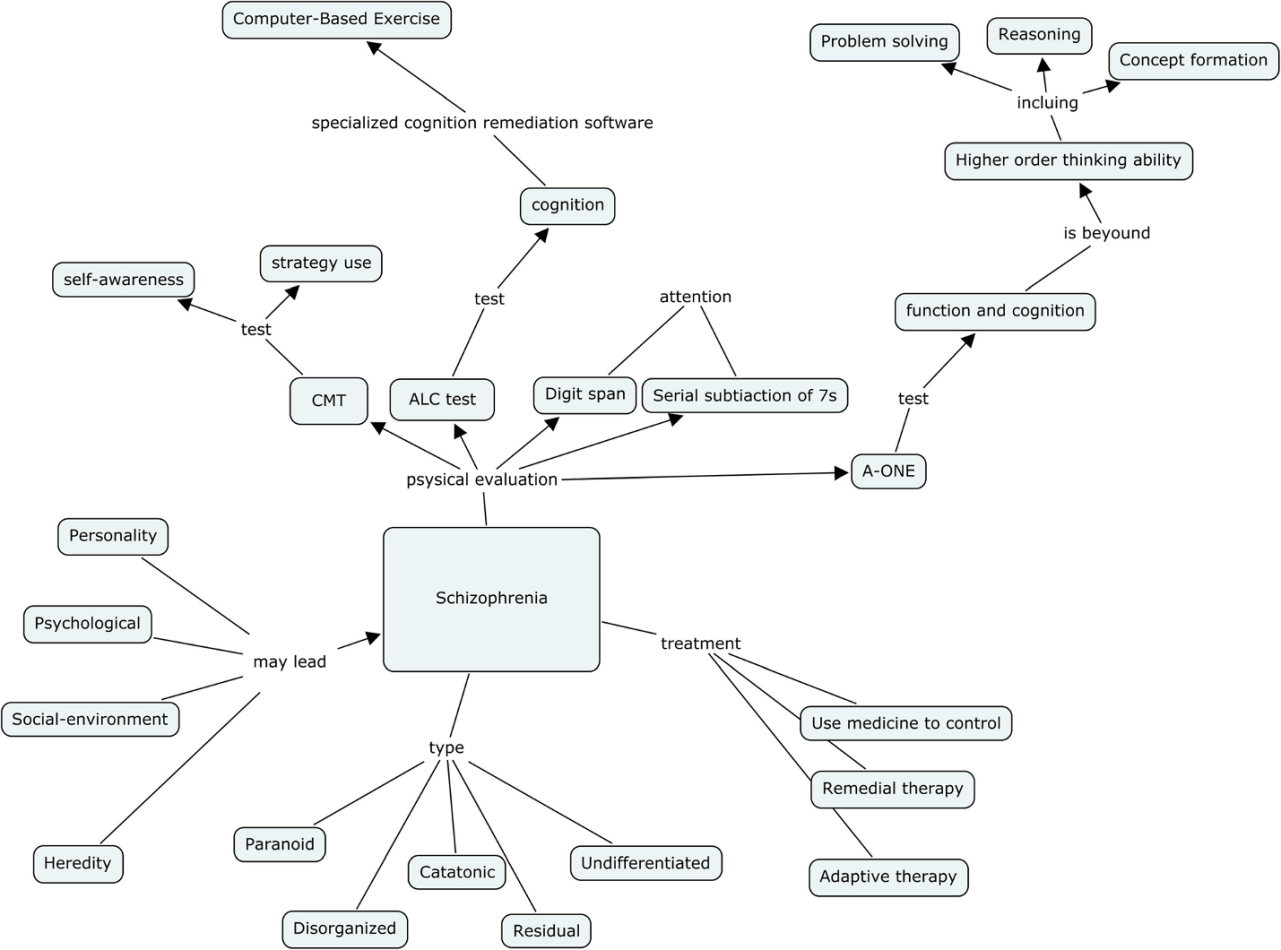
An example of isolated concept mapping

**Isolated mapping** was typical, with several single concepts linked to the main map without reference to other associated concepts. The concepts were sometimes misplaced in the hierarchy and seemed to float outside of the main map with an arrow in the opposite direction. In addition, the concepts were less inclusive and difficult to accommodate in the knowledge structure. Figure 2 shows an isolated concept map from the LBL group. Note the morphology of the map, with four concepts on the left side connected to the core concept; however, the connecting arrow is in the opposite direction. This revealed that some concepts were isolated and difficult to progressively differentiate from a superordinate concept, and then connected to the knowledge structure. According to our analysis (Table 2), 15.4 % of the LBL and 7.7 % of the PBL students’ first maps were sorted into this category. In the second drawing, 11.5 % of LBL students, and no PBL students, still produced isolated maps.

**Table 2:** Summary of chi square tests for the test of the homogeneity of the three concept mapping categories in the two groups

		Isolated		Departmental		Integrated			
Groups		n	%	n	%	n	%	χ^2^(2)	*p*
PBL	1st	2	7.7	0	0.0	24	92.3	6.84	0.033^*^
	2nd	0	0.0	4	15.4	22	84.6		
LBL	1st	4	15.4	4	15.4	18	70.4		
	2nd	3	11.54	6	23.1	17	64.7		

*p < 0.05; Isolated = isolated mappings, Departmental = departmental mappings, Integrated = integrated mappings; PBL = problem-based learning group, LBL = lecture-based learning group

**Fig. 3:**
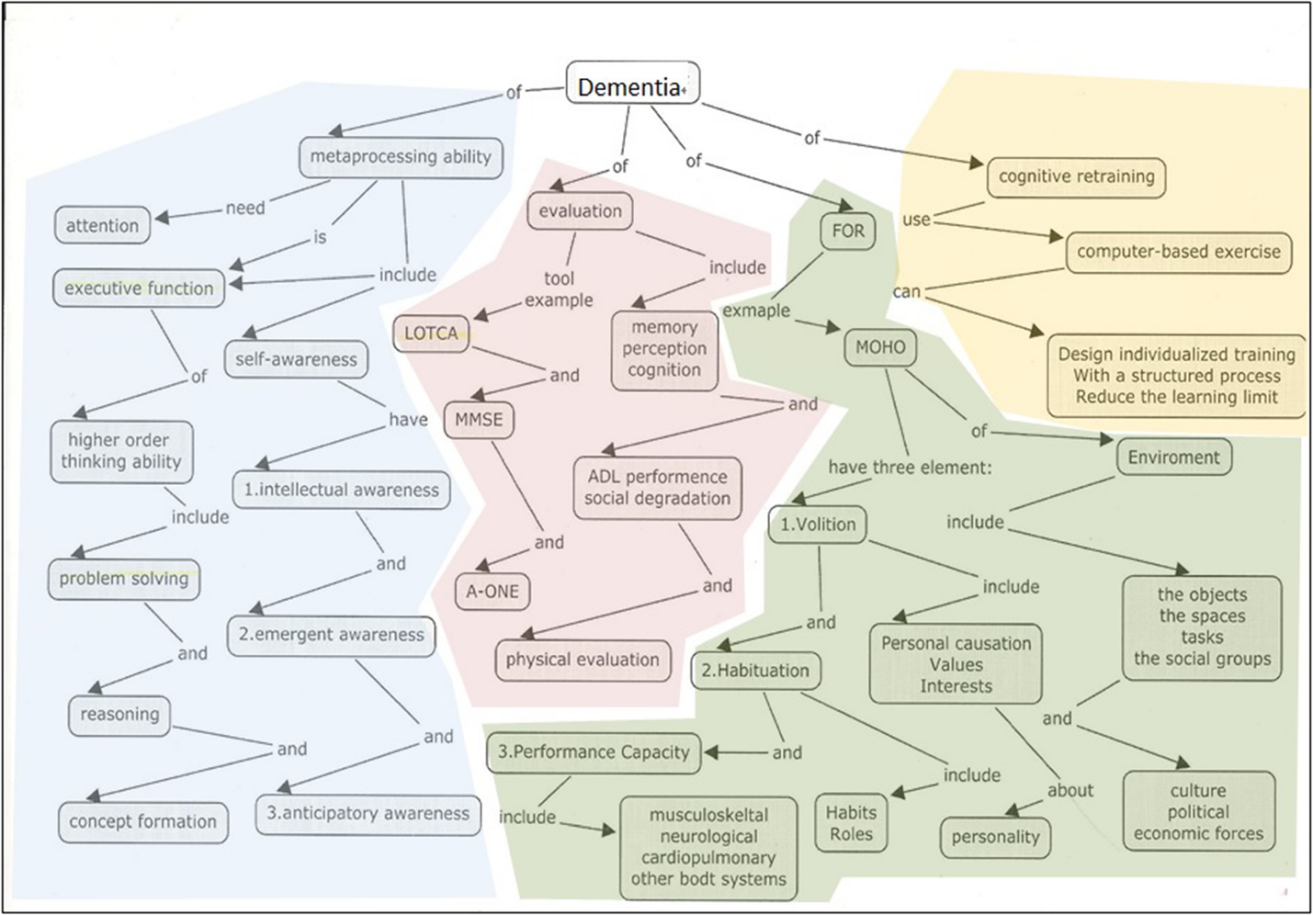
An example of departmental concept mapping

**Departmental mapping** refers to maps with several separated units or micro maps connected by a single arrow to superordinate concepts. A lack of cross-linkages among micro maps was characteristic of this category, in which the relationships among those micro maps could not be identified and a network-like map was not established. Figure 3 shows a departmental concept map composed of four micro maps; however, there are no cross-linkages to connect them. This reveals that acquired knowledge was included in the separate blocks; however, due to the lack of horizontal links, these concepts could not supplement one another or be mutually retrieved when utilized in a problem-solving task. In contrast to the isolated maps that often appeared in the first drawings, departmental maps were more common in the second drawings. For the first maps, 15.4 % from the LBL group and none from the PBL group were sorted as departmental maps. For the second maps, 23.1 % from the LBL group and 15.4 % from the PBL group were in this category (Table 2).

**Fig. 4:**
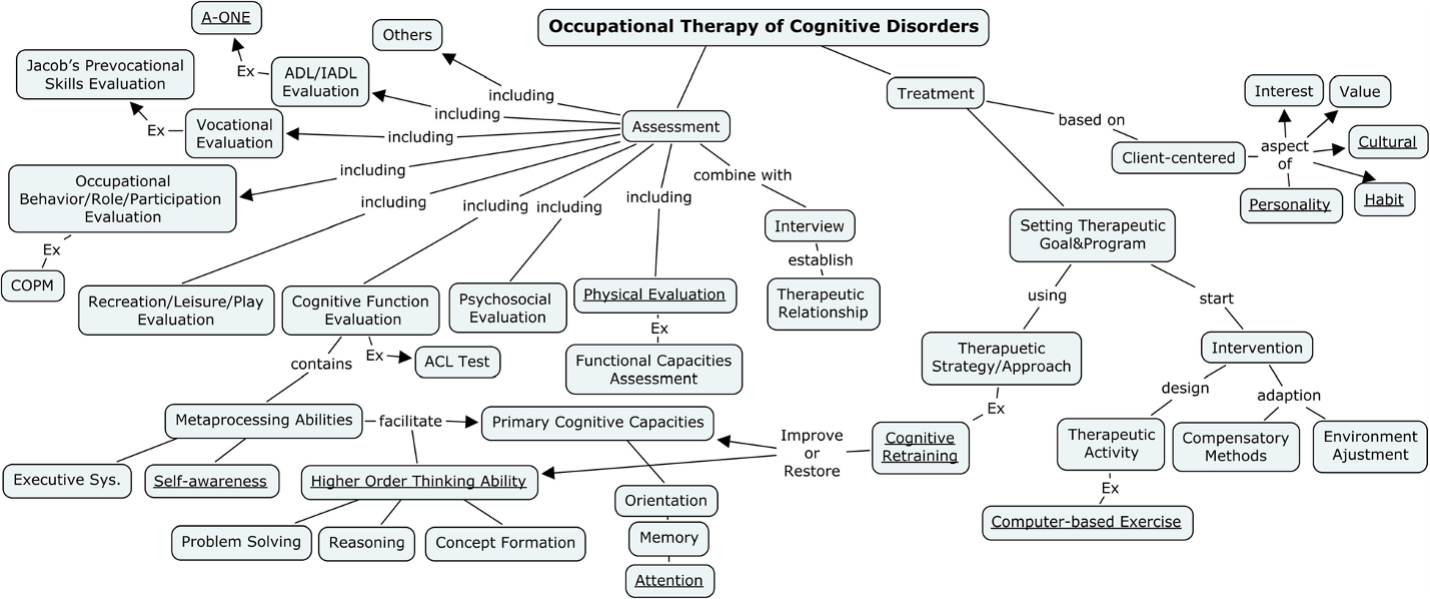
An example of integrated concept mapping

**Integrated mapping** demonstrates a good integration of concept mapping in which a superordinate concept appears at the highest level and then cross-linkages among various segments of knowledge are established to illustrate how these micro maps are related to one another. Figure 4 shows an integrated concept map which has a superordinate core concept, “Occupational Therapy of Cognitive Disability,” and then branches into two micro maps, “Assessment” and “Treatment.” Note the complexity; the map contains sufficient concept relationships to illustrate the theme, but it also has a reasonable hierarchy to show progress in the differentiation of the knowledge structure, as well as cross-linkages between and within the micro maps. Therefore, this map can be identified as a truly integrated concept map. Based on the analysis, 92.3 % of the first and 84.6 % of the second drawings of the PBL group were integrated maps, and 70.4 % of the first and 66.7 % of the second drawings of the LBL group were sorted into this category. The data indicated a trend in the PBL and LBL groups: A number of concepts were imported, the percentage of isolated and integrated maps declined, and the percentage of departmental maps increased. A test of homogeneity among the three concept mapping categories in the two groups (Table 2) showed that the two groups exhibited differences in terms of the concept mapping categories; that is, there was no homogeneity in the changes that took place.

### Analysis of the structure of concept mapping

Table 3 shows the means and SDs of the PBL and LBL groups for the four scoring parameters. The average of the PBL group was significantly higher than that of the LBL group. Independent-samples *t*-tests show significant differences between the PBL and LBL groups in terms of the relationships among the concepts (*t* (50) = 2.93, *p* =0.005; *d* = 0.81; power =0.93), hierarchy levels (*t* (50) = 2.25, *p* =0.029; *d* = 0.61; power =0.86), and cross linkages (*t* (50) = 2.30, *p* =0.026; *d* = 0.62; power =0.87). Although the mean of “examples” was higher in the PBL group, the difference was not statistically significant (*t* (50) = 1.14, *p* =0.26). The coefficients of Cohen’s *d* showed a medium to large effect size in the three parameters, and the power ranged from 0.86 to 0.93.

**Table 3:** The *t*-test summary of concept mapping scoring levels in the PBL and LBL groups (N = 52)

	PBL (n = 26)		LBL (n = 26)		*t(50)*	*p*	Cohen’s *d*	power^a^
Parameters	*M*	*SD*	*M*	*SD*				
Concept relationships	116.5	45.29	81.7	40.84	2.93	0.005^*^	0.81	0.93
Hierarchy levels	17.0	3.16	14.3	5.42	2.25	0.029^*^	0.61	0.86
Cross linkages	9.3	8.23	5.1	4.76	2.30	0.026^*^	0.62	0.87
Examples	8.9	8.17	6.0	9.73	1.14	0.261	0.33	0.26

*p < 0.05; PBL = problem-based learning, LBL = lecture-based learning; *M* = mean; *SD* = standard deviation

^a^Post-hoc power analysis

## Discussion

The present study aimed to understand the effects of PBL on students’ knowledge structures as demonstrated by the patterns of and differences among concept maps. The central result of this study was that most of the concept maps in the PBL group eventually exhibited the integrated concept mapping pattern, which is an identifying mark of a high-quality knowledge structure. The scores on the parameters of concept mapping networks were higher in the PBL group than in the LBL group. The results of the analysis indicated that there were important properties of PBL that contributed to the students’ learning with regard to the knowledge structure.

### Learning situations trigger motivation and enhance the acquisition of knowledge

Two students in the isolated map category and two students in the integrated map category in the PBL group moved into the departmental map category on the second drawing, while one student in the isolated category and one in the integrated category in the LBL group moved into the departmental category on the second drawing. Although it seemed that students performed almost the same in the PBL and LBL groups, the distribution of the three categories of concept mapping in the two groups showed that this was not the case. A close look at the chi-square test for homogeneity revealed that PBL improved the students’ performances more than did LBL. The findings demonstrated that the PBL property of situational learning with more clues helped to create a desire in students to find out more about the topic and to make that information meaningful. As a result, they were continuously expanding the limits of their knowledge and incorporating new and relevant concepts into their knowledge structures [13]. In contrast, de-contextualized learning such as LBL may result in the compartmentalization of concepts or propositions. Although these students tried to connect new concepts to the main map, they were unable to develop a map with an integrated knowledge structure due to the few cues and reminders provided during the learning process.

### PBL facilitates connecting knowledge during cognitive construction

Although both student groups were engaged in learning over the same period of time, the performance of the PBL group with regard to the concepts of relationships, hierarchy levels, and cross linkages was significantly better than that of the LBL group. This difference could be a result of PBL on the activation and elaboration of previously learned knowledge, whereby the students’ reasoning skills might be enhanced. Knowledge is the underpinning of operational and thinking skills; thus, there is no skill without knowledge. In a clinical task requiring a large amount of knowledge, and especially integrated knowledge, it is suggested that concept mapping can help with visualization of the thinking process [13].

### Problem space enhances conceptual differentiation and integration

According to Ausubel’s meaningful learning theory, PBL provides a holistic perspective and valid problem space for students to enhance their knowledge structure through gradual progressive differentiation and integrated reconciliation [31]. Lecture-based teaching often lacks strategies to integrate the knowledge structure and results in isolated or compartmentalized mapping. In addition, it is also important to note the function of group dynamics in PBL. PBL provides opportunities for students to present their own learning experiences and to value their peers’ perspectives; all of these experiences could help them to construct their own frameworks [26]. This may also explain why all of the scoring parameters were higher in the PBL group.

In the current study, isolated and departmental maps continued to appear in the learning process in both groups. This may be a typical result stemming from the large influx of concepts in a short learning period in both the problem scenarios and traditional lectures. As medical education emphasizes the connections and relationships between basic science and clinical knowledge, the amount of learning materials handed to the students should be given careful consideration so that the students have enough time to develop an integrated knowledge structure.

## Conclusions

This study identified three categories of concept mapping: isolated, departmental, and integrated. It appears that PBL can help students engage in integrated concept mapping and achieve a more integrated knowledge structure. The findings also revealed that the effect of PBL on the acquisition and integration of knowledge was robust. In order to solve problems in PBL, students connect descriptive knowledge with procedural knowledge and create more details and cross linkages in their knowledge structures, which will benefit clinical reasoning in the future. The findings of this study suggest that educators aiming to enhance their students’ knowledge structures should incorporate PBL and concept mapping in the curriculum.

### Limitations

Although the results of the study revealed the benefit of using concept mapping to discover the knowledge structure in PBL, our design bears the inherent limitations of the learning materials and the learning time of the students. The study clearly lacked explanations for the amount of learning materials relative to cognitive loading, and it did not explore the long term effect of concept mapping coupled with PBL. These are important issues that deserve to be addressed and explored further.

## Abbreviations

PBL: Problem-based learning
LBL: Lecture-based learning
